# Evaluation of antibacterial and antioxidant activities of Sesame (*Sesamum indicum*) meal protein hydrolysate produced by *Bacillus coagulans* (IBRC 10807) fermentation

**DOI:** 10.1038/s41598-025-21765-1

**Published:** 2025-10-29

**Authors:** Parisa Raei, Morteza Khomeiri, Alireza Sadeghi Mahoonak, Ali Moayedi, Mahboobeh Kashiri

**Affiliations:** Department of Food Science and Technology, Gorgan University of Agricultural Sciences and Natural Resources, Gorgan, Iran

**Keywords:** Sesame protein hydrolysate, Antimicrobial, Antioxidant, B. coagulans, FTIR, Biochemistry, Biotechnology, Microbiology

## Abstract

In this research, sesame meal protein hydrolysate (SPH) was obtained from the sesame protein after hydrolysis by *Bacillus coagulans*. At first, the peptide concentration test was performed at different times to confirm the protease activity of *B. coagulans*. The chemical composition and total amino acid contents of sesame meal were determined. Fermentation conditions were optimized using response surface methodology (RSM). The results showed DPPH radical scavenging up to 70%, reducing power up to 0.779, and inhibitory activity against *Staphylococcus aureus* up to 78%, *Escherichia coli* up to 60%, *Listeria monocytogenes* up to 80% and *Clostridium perfringens* up to 85%. The antioxidant activity of the optimal sample was investigated at concentrations ranging from 10 to 50 mg/mL. The results demonstrated that 50 mg/mL of the SPH had the highest antioxidant activity. Analysis of amino acids by HPLC revealed that glutamic acid and glycine had the highest concentrations, and all essential amino acids were detected. SEM analysis showed smaller heterogeneous particles of protein hydrolysate, which confirmed the hydrolysis process during fermentation. FTIR results showed that different functional groups were formed, which confirmed the hydrolysis of sesame protein. Generally, sesame protein can be a good source in the fermentation system by *B. coagulans* to produce hydrolysate with antimicrobial and antioxidant properties.

## Introduction

Nowadays, due to the harms such as increasing the possibility of heart and carcinogenic risks and the occurrence of digestive problems that synthetic food additives have for the body, using natural preservatives is an important approach for extending the shelf life of food^1^. Chemical preservatives are frequently utilized in the food industry due to their simple process and affordable cost. However, problems have been caused due to the inadequate utilization of chemical preservatives^2^.

Natural preservatives have been described as potential alternatives to chemical preservatives due to their antioxidant or antibacterial properties^3^. There is an increasing attention on antimicrobial compounds derived from plants and animals that can efficiently control microbiological and chemical spoilage, hence improving the shelf life of food^4^. So far, many synthetic antioxidants have been used in the food industry, including antioxidants such as BHA and BHT, which have strong antioxidative properties^5^. However, the interest of consumers to use natural products has increased the attention of the food industry toward the use of natural antioxidants in order to extend the shelf life or enhance the safety of food^6^.

Bioactive peptides and protein hydrolysates derived from food have antimicrobial and antioxidant activity. As bioactive food additives extend the shelf life of food products, they have gained significant attention in the food industry^3^. Enzymatic hydrolysis and fermentation are used to produce peptides from proteins. *Bacillus* species can produce significant amounts of non-specific proteolytic enzymes and have high growth potential in harsh environments with limited and inexpensive carbon and nitrogen sources. Therefore, the fermentation process is more cost-effective than hydrolysis by commercial enzymes^7^. *Bacillus* strains, including *B. licheniformis*^8^, *B. subtilis*^9^, *B. altitudinis* HK02^10^, and *B. mojavensis* A21^11^ were used as fermenting bacteria that are highly efficient in protein hydrolysis to produce bioactive peptides. Bioactive peptides can be produced from various plant and animal sources. Plants are a rich source of proteins with high nutritional value. In recent studies, agricultural and industrial waste proteins have been used to produce peptides with bioactive properties^12^. Sesame (*Sesamum indicum L*.) is a significant oilseed plant with global importance, commonly used for various purposes. It is an important source of nutrients and energy. It is reported that sesame seeds contain 50–60% oil and 25% protein^13^. Sesame meal is a protein-rich by-product obtained after extracting oil from sesame seed, containing roughly 50% protein, which is used as a high-protein food source for livestock and poultry^14^. The researchers reported that the peptide obtained from the enzymatic hydrolysis of sesame protein by alcalase and trypsin had an antioxidant effect; these researchers identified seven peptides, determined their sequence, and demonstrated that it has the highest antioxidant activity, and it can be used as functional foods^15^.

The purpose of this research was to optimize the fermentation conditions containing time, temperature, and protein concentration for the preparation of sesame meal protein hydrolysate by *B. coagulans* and to investigate the antimicrobial activity against pathogenic bacteria and antioxidant properties, including DPPH radical inhibition and ferric ion reducing power. Chemical composition, amino acid profiles, Fourier transform infrared spectroscopy, and the effect of different concentrations of optimal samples on antioxidant activity were evaluated.

## Materials and methods

### Materials

Sesame meal was purchased from the Keshavarz Food Industry CO (Qom, Iran). 2, 2-diphenyl-1-picrylhydrazyl (DPPH) and o-phthaldialdehyde (OPA) were prepared from Sigma Aldrich (Darmstadt, Germany).

#### Microbial strains

*B. coagulans* IBRC 10,807, *E. coli* PTCC1399, *C. perfringens* PTCC 1765, *L. monocytogenes* PTCC 1298, *S. aureus* PTCC 29,213.

### Preparation of Sesame meal protein isolate

At first, sesame meal was ground, then defatted using hexane (1:5 w/v) and was agitated at ambient temperature for 3 h. Hexane was removed using a Buchner funnel, then dried in an air-drying oven at 30 °C and passed through a 40-mesh sieve. The defatted sesame meal powder was blended with deionized water (1:10 ratio w/v), and the pH value was increased to 11 using 1 N NaOH, and the mixture was agitated continuously for 1 h at 50 °C. It was centrifuged (Combi-514R, South Korea) at 6000 rpm for 20 min to remove the insoluble fraction. Next, the pH of the supernatant was altered to 4.2 (isoelectric pH) with 1 N HCl and stirred (Labtron, Iran) for 30 min. The precipitate obtained was centrifuged at 8000 rpm for 20 min, and the protein isolate was kept for further experiments^15^.

### Chemical composition

The chemical composition of raw sesame meal, defatting, and protein isolate containing moisture, protein content, fat, and ash content were estimated by the American Association of Cereal Chemists^16^.

### Amino acid composition (by HPLC)

Analysis of amino acids was performed by HPLC (Young Lin Korea) and Teknokroma RP-C18 ODS-A column (15 cm × 0.46 cm, 5 μm), flow rate (1.3 mL/min) and temperature of 35 °C, using the method of Jiang et al.^17^. Sesame protein was hydrolyzed by 6 N HCl at 110 °C for 24 h. The amino acid contents were stated as g/100 g of the total amount of amino acids.

### Peptide concentration

Peptide concentration of sesame protein hydrolysate was evaluated based on the reaction of the amino groups using the o-phthalaldehyde (OPA) reagent. 2.5 mL of the supernatant and 5 mL of 10% TCA were mixed, and after 10 min, it was shaken for 10 min at 5000 rpm. The supernatant obtained was used to evaluate proteolysis. 3 mL of prepared OPA reagent was combined with 200 µL of supernatant, and after 2 min, the absorbance was obtained at 340 nm. A standard curve of different concentrations of L-serine amino acid (0.002-0.5 mg/mL) was prepared for calculating protease activity^18^.

#### Optimization of fermentation

Response surface methodology (RSM) was determined to optimize the temperature, time, and substrate concentration. The central composition design was used with three independent variables: time, temperature, and substrate concentration. The levels of temperature, time, and substrate concentration (SPI) are given in Table 1. 0.03% MgSO_4_, 0.1% K_2_HPO_4_, 1% glucose, 0.05% CaCl_2_ w/v, and 2% v/v of the inoculum were added to the culture medium. The supernatant obtained after fermentation was filtered with a 0.45 μm filter, then the pH was increased to 7. The supernatants were freeze-dried (FDB 5503, South Korea) and stored at -20 °C. Antimicrobial and antioxidant tests (DPPH radical scavenging and reducing power) were performed as a response variable^19^.

**Table 1:** Independent variables and coded levels central composite Design.

Factor	Independent variables	Units	Coded levels				
			−α	−1	0	+ 1	+α
A	temperature	°C	24.89	30.00	37.5	45.00	50.11
B	time	h	3.82	12.00	24	36.00	44.18
C	concentration	%	0.6364	2.00	4	6.00	7.36

### Antimicrobial activity

The antimicrobial activity of SPH was done based on the method of Aguilar-Toalá et al.^20^. *E. coli*, *S. aureus*, *L. monocytogenes*, and *C. perfringens* were used to analyze antimicrobial activity. 20 mg of SPH was dissolved in 900 µL of Mueller Hinton Broth culture medium, and 100 µL of bacteria with a concentration of 10^5^ cfu/mL was mixed and incubated at 37 °C for 24 h. Absorbance was measured at a wavelength of 600 nm. Muller Hinton broth culture medium containing bacterial suspension (10^5^ cfu/mL) was used as a positive control.

### Antioxidant activities

#### DPPH radical inhibition

DPPH radical scavenging activity of sesame protein hydrolysate was evaluated using the method defined by Jemil et al.^21^. 500 µL of SPH was combined with 375 µL of ethanol. Afterwards, 125 µL of 0.15 mM DPPH (prepared in absolute ethanol) was added to the sample and left in the dark for 30 min. The absorbance of the samples was performed at 517 nm. Distilled water was used instead of the SPH in the negative control. The percentage of inhibition was determined by the following equation:

DPPH radical scavenging: $\:\:\frac{\text{A}-\text{B}}{\text{B}}\times\:100$%. A represents the absorbance value of the control, B represents the absorbance value of the solution.

#### Reducing power

The reducing power of the SPH was performed by the method of Bougatef et al.^22^. 500 µL of SPH with phosphate buffer and 500 µL of potassium ferricyanide were combined and incubated at 50 °C for 30 min. Afterward, 10% TCA was applied to the solution. Then, the mixture was shaken at 12,000 rpm for 10 min. The obtained supernatant was mixed with 0.1% w/v ferric chloride. The absorbance of the mixture was determined at a wavelength of 700 nm after 10 min.

#### Scanning electron microscopy

SEM was conducted using a scanning electron microscopy (Tescan, Czech Republic). Sputtering was used to apply a thin layer of gold to the samples. The images were observed at a working distance of 4.95 mm and an accelerating voltage of 15 kV^23^.

#### Fourier transform infrared spectroscopy

Fourier transform infrared spectroscopy (Perkin Elmer, Spectrum RX1) of SPI and SPH was examined in the wavelength range of 400–4000 cm^− 1^ with a resolution of 4 cm^− 1^. Sample preparation for analysis was accomplished by mixing the sample and potassium bromide (KBr) powder^24^.

### Statistical analysis

In this research, the response surface methodology (RSM), based on the central-composite design, was used to determine optimal levels of the independent variable. The statistical data analysis was executed using the Stat-Ease Design Expert software version 11. SPSS 26 statistical software was utilized to analyze the chemical composition, protease activity, and antioxidant activity. The Duncan’s test was conducted to compare the means at the significance level of 5%.

## Results and discussion

### Chemical composition

The range of moisture, protein, fat, and ash of raw meal, defatted sesame, and protein isolate is given in Table 2. According to the results obtained, the fat content of raw sesame meal is reduced from 16% to 2.33% in protein isolate. The final reduction of fat in protein isolate was due to using hexane solvent, protein extraction in an alkaline solution, and protein precipitation at the isoelectric point^25^. The lowest amount of ash was related to protein isolate due to the removal of large amounts of non-protein compounds in the protein extraction process. The protein content of raw and defatted sesame meal was 27.3% and 42.63%, respectively, indicating that sesame meal is a rich source of protein that can be used as a protein-rich substrate^26^.

**Table 2:** The chemical composition of Raw Sesame meal, defatted Sesame meal, and protein hydrolysate.

	Fat %	Protein %	Moisture %	Ash %
Raw sesame meal	16.2 ± 0.17^a^	27.3 ± 0.3^c^	9.54 ± 0.05^a^	6.25 ± 0.1^b^
Defatted sesame meal	4.2 ± 0.17^b^	42.67 ± 0.35^b^	7.13 ± 0.3^b^	7.5 ± 0.1^a^
Sesame protein isolate	2.33 ± 0.57^c^	91.1 ± 0.69^a^	1.86 ± 0.05^c^	0.76 ± 0.05^c^

### Sesame amino acid profile

**Fig. 1:**
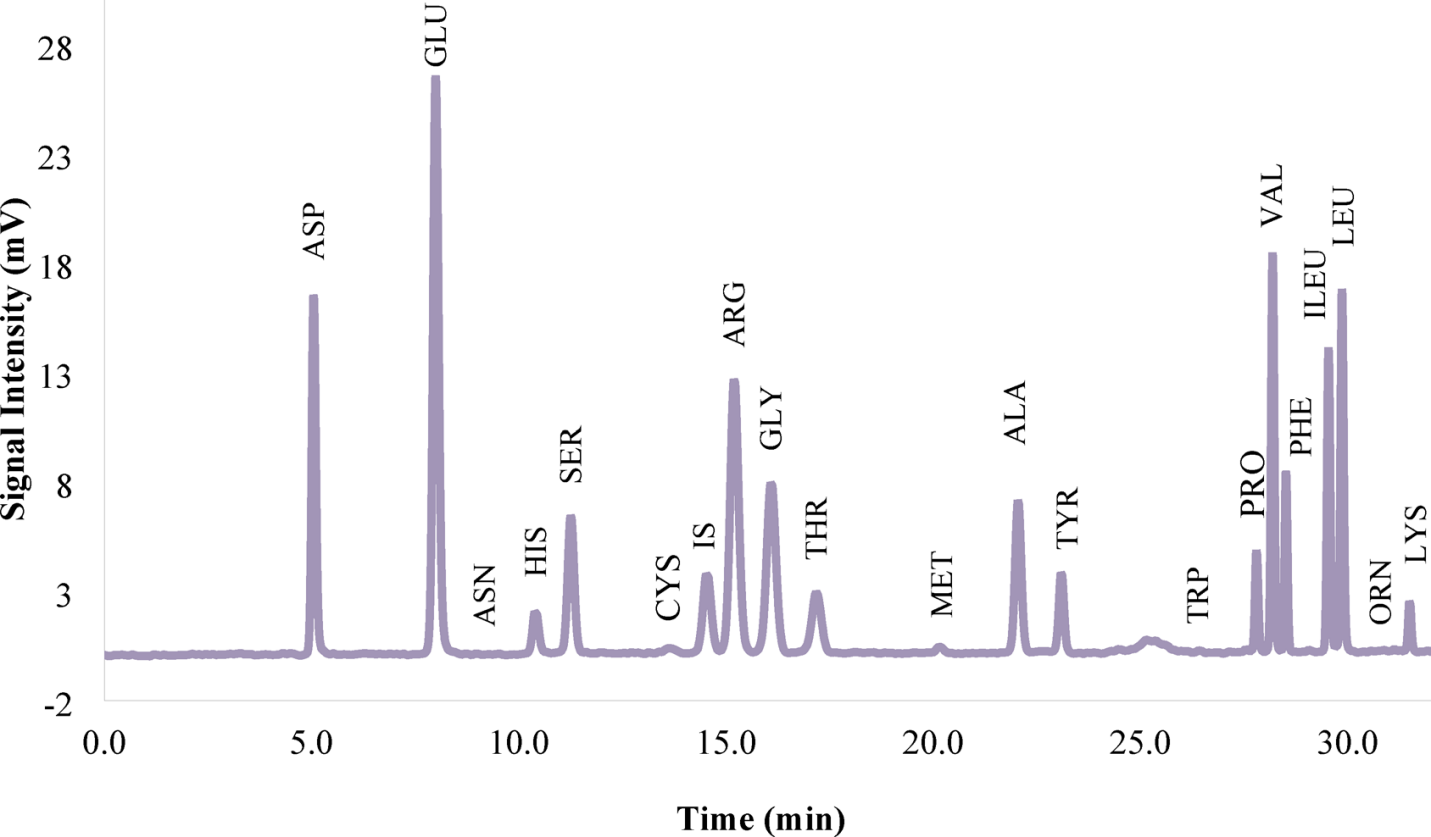
HPLC chromatogram displaying the amino acid composition of sesame protein isolate (SPI).

​The functional and physicochemical properties of sesame protein and its use as a food supplement for nutritional applications have been reported by Achouri et al.^25^. According to Table 3; Fig. 1, the total amount of amino acids was 92.31 g/100 g. Glutamic acid 18.26, arginine 12.39, and aspartic acid 9.34 g/100 g had the highest amounts^27^. Among them, some of the essential amino acids have been reported, including phenylalanine 4.93, arginine 12.39, valine 6.19, lysine 1.82, leucine 7.98, isoleucine 4.66, histidine 2.13, threonine 3.14 and methionine 1.72 g/100 g; consequently, 44.96 g of the total amount of amino acids were related to these amino acids. Onsaard^28^ reported that sesame protein isolate has high amounts of the amino acids arginine, glutamic acid, leucine, and valine, which can be used as a dietary supplement. The difference in the amount of amino acids can be attributed to genetic differences between sesame varieties^29^. Hydrophobic amino acids, including phenylalanine, proline, methionine, alanine, leucine, isoleucine, tyrosine, and lysine, which are reported in Table 3, are responsible for the bioactive properties of protein hydrolysate. Hydrophobic amino acids with antioxidant properties bind to free radicals, consequently limiting their availability to the target cell^30^. Amino acids such as tryptophan, tyrosine, cysteine, methionine, lysine, histidine, and arginine play an important role in the antimicrobial properties of protein hydrolysate. The binding of antimicrobial protein hydrolysate to the cell membrane causes disruption of membrane integrity, depolarization, and osmotic imbalance, ultimately leading to cell death^31^. The findings demonstrate that sesame meal can be used as a suitable protein source for producing protein hydrolysates with bioactive properties.

**Table 3:** The amino acid composition of Sesame protein isolate.

Amino acid	Result (g/100 g)
Aspartic acid	9.34
Glutamic acid	18.26
Asparagine	ND*
Histidine	2.13
Serine	3.52
Cysteine	0.9
Glutamine	ND*
Arginine	12.39
Citruline	ND*
Glycine	3.91
Threonine	3.14
Methionine	1.72
Alanine	3.95
Tyrosine	3.21
Prolin	3.21
Valine	6.19
Phenylalanine	4.93
Isoleucine	4.66
Leucine	7.98
Ornithine	ND*
Lysine	1.82
Tryptophan	1.07
Total	92.31

ND*: <0.05 g/100 g.

### Peptide concentration

**Fig. 2:**
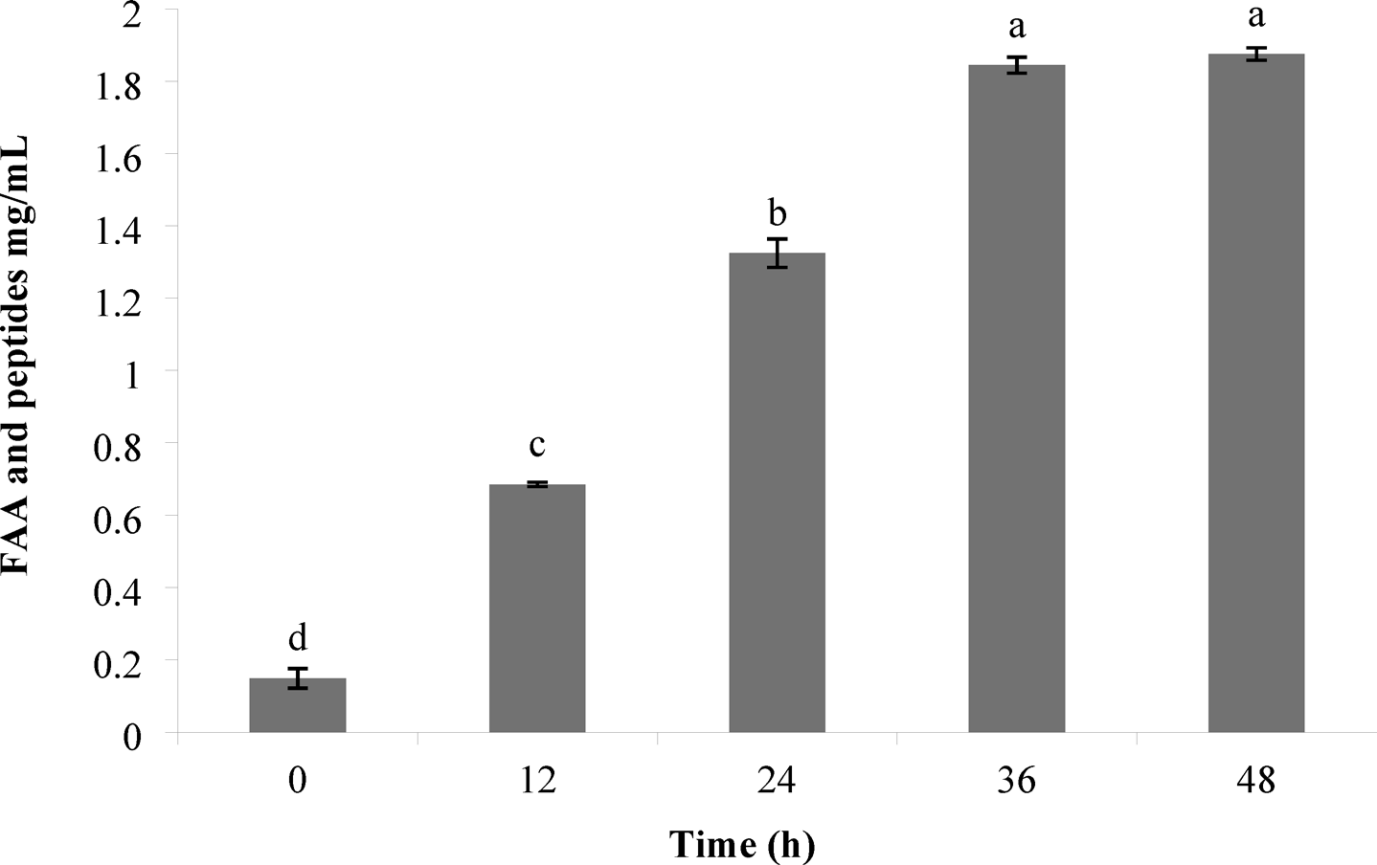
Analysis of the amino acid and peptide concentrations of hydrolysates at different time intervals. Data values were performed in triplicate (Mean ± SD).

Evaluating proteolysis activity is a crucial parameter to examine the release of peptides in the fermentation process. In addition, the peptide concentration was evaluated using the OPA method. This method’s basis was measuring the free amino group produced during the fermentation process. The results in Fig. 2 demonstrated that the progress of fermented hydrolysis of protein during 0, 12, 24, 36, and 48 h after fermentation for this strain was significant (*p* < 0.05). The hydrolysis process showed that *B. coagulans* has used SPI as a carbon and nitrogen source and has obtained its nutritional needs. The peptide concentration exhibited a rapid increase after 12 h of fermentation, and this process persisted until 48 h. The highest peptide concentration was found at 36 h after fermentation. Some peptides were observed in the culture medium before fermentation, probably released during sterilization^32^. With the increase in time up to 36 h, the content of amino acids and peptides increased continuously, and then at 48 h, no significant increase was observed (*p* > 0.05). Li & Wang reported that during fermentation of chickpeas by *B. subtilis* lwo, increasing protease activity led to an increased release of peptides and free amino nitrogen, which attained their highest values^33^. Proteolysis of cereal protein and plant seeds by *Bacillus* species has been reported. Zhu et al.^34^ reported that *B. subtilis* showed the highest protease activity after 24 h of fermentation in the presence of soybean substrate. According to the findings of Shi et al.^35^ soybean and corn meal fermented by *B. subtilis* were hydrolyzed to peptides less than 25 kDa.

### Optimization of fermentation conditions

**Fig. 3:**
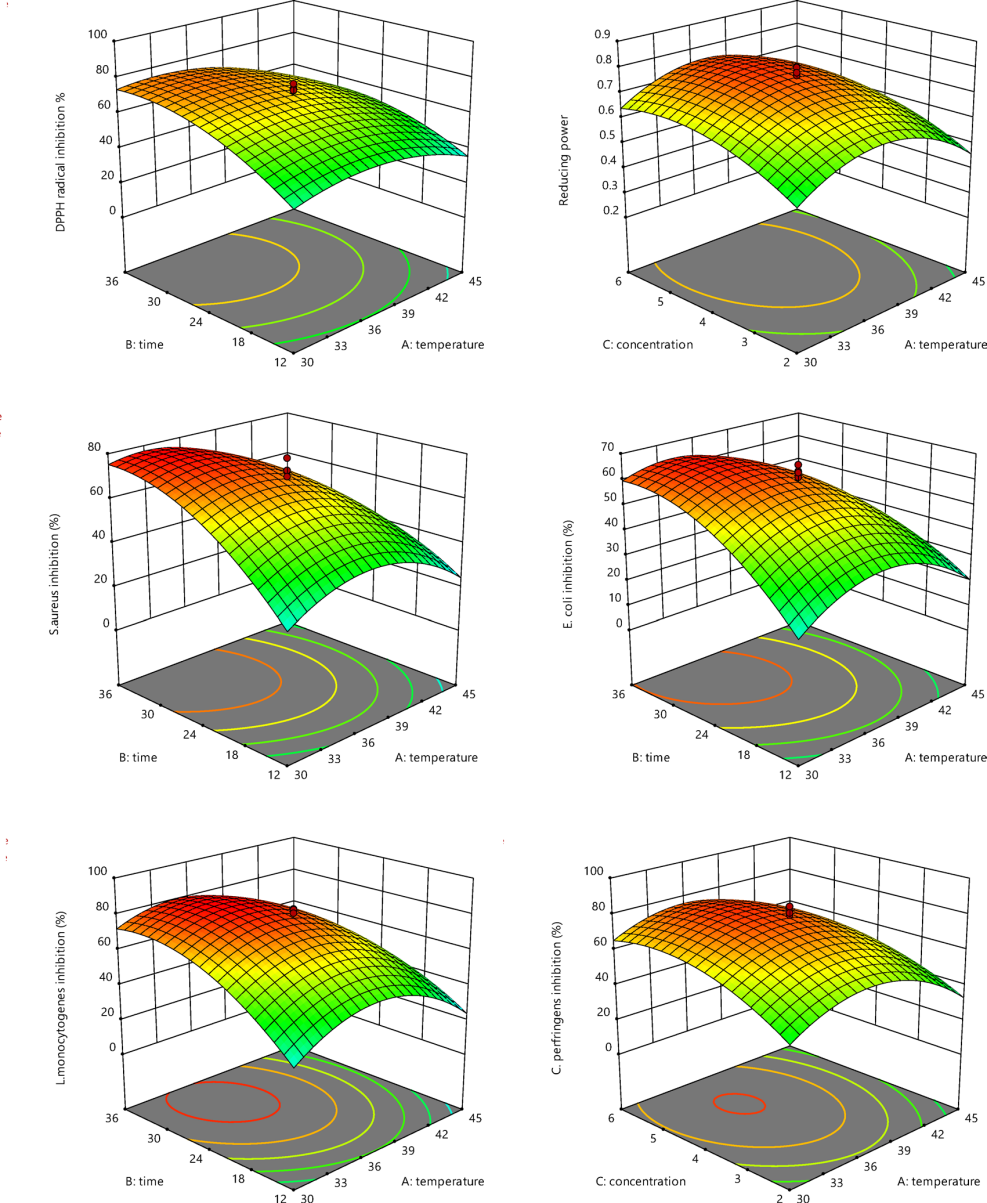
Contour plots demonstrating the effects of independent parameters (temperature, time, and SPH concentrations) on antibacterial and antioxidant activities.

In this research, independent variables included substrate concentration, temperature, time, and the effect of these factors on dependent variables, including DPPH radical inhibition, ferric ion reducing power, and the antimicrobial properties of hydrolysate against *E. coli*, *S. aureus*, *C. perfringens*, and *L. monocytogenes* were evaluated. The results are given in Table 4. The highest DPPH radical inhibition and reducing power levels were 83.7% and 0.799% (absorbance at 700 nm), respectively. A polynomial regression model with the quartic term (Eqs. 1, 2) was used to analyze DPPH radical scavenging and reducing power. According to the results of the analysis of variance in Table 5; Fig. 3, the effect of temperature, time, and A^2^ and B^2^ variables on DPPH radical inhibition and the effect of time, substrate concentration, and A^2^, B^2^, and C^2^ variables on reducing power were significant (*p* < 0.05). The regression model with R^2^ of 0.8106 and R^2^ of 0.8649 was proposed for DPPH radical scavenging and reducing power, respectively. Therefore, the model could explain 81.06% and 86.49% of the total variations in the investigated variables.

**Table 4:** The matrix of the central composite design and different responses (antioxidant and antimicrobial activity).

Run	A: temperature (°C)	B: time (h)	C: substrate concentration (%W/V)	DPPH radical scavenging (%)	Reducing power	Inhibition of S. aureus (%)	Inhibition of E. coli (%)	Inhibition of L. monocytogenes (%)	Inhibition of C. perfringens (%)
1	30	36	2	66.6	0.486	60.7	40.3	50.6	46.3
2	37.5	24	0.636	46.3	0.345	42	33	38	36.6
3	37.5	24	7.363	83.7	0.76	76.9	66	79.6	87.7
4	37.5	3.818	4	18.6	0.276	9.8	10.8	8.6	5.6
5	37.5	24	4	76.6	0.779	68.8	62.7	80.7	84.4
6	37.5	24	4	74	0.77	72	60.9	83	79.9
7	45	12	2	43.2	0.42	14.5	11.2	9.5	13.8
8	37.5	24	4	72.8	0.768	78	42	78	80.8
9	45	36	6	38.9	0.513	18	12.8	15.6	24.4
10	30	12	6	38.3	0.411	24.6	18	22	33
11	37.5	44.18	4	78	0.682	74.4	61.8	85	86
12	45	36	2	52.6	0.382	22.5	12.8	19.6	28
13	50.11	24	4	30.8	0.312	12.8	10.6	8.7	24.3
14	30	12	2	45.8	0.363	19.8	16.4	21.8	18.6
15	37.5	24	4	56	0.65	52	58.9	82.3	82.7
16	30	36	6	67.3	0.511	65.3	49.3	56	60.4
17	24.88	24	4	57.8	0.459	40.8	30.7	38	42
18	37.5	24	4	73	0.799	69.9	65.7	59.9	60
19	37.5	24	4	69.9	0.802	72.6	63.2	82.6	79.6
20	45	12	6	28.8	0.336	9.6	10.4	12.7	19.2

**Table 5:** Analysis of variance for antimicrobial and antioxidant properties of Sesame protein hydrolysate (SPH).

	DPPH radical scavenging %	Reducing power	Inhibition of S. aureus %	Inhibition of E. coli %	Inhibition of L .monocytogenes %	Inhibition of C .perfringens%
P-value						
Model	0.0140***	0.0025***	0.0011***	0.0058***	0.0019***	0.0044***
A: temperature	0.0421***	0.3284	0.0068***	0.0413**	0.0266**	0.1018
B: time	0.0019***	0.0152***	0.0008***	0.0073***	0.0039***	0.0039***
C: concentration	0.6709	0.0355***	0.2219	0.1972	0.2020	0.0687**
AB	0.3808	0.7651	0.0880**	0.1873	0.2621	0.4322
AC	0.5241	0.9261	0.5976	0.7591	0.8817	0.5541
BC	0.7843	0.4987	0.9955	0.8252	0.9629	0.8354
A²	0.0082***	0.0003***	0.0003***	0.0006***	0.0002***	0.0009***
B²	0.0317***	0.0019***	0.0057***	0.0177***	0.0048***	0.0058***
C²	0.2510	0.0143***	0.1031	0.0913**	0.0303***	0.0568**
Lack of fit	0.0957	0.0534	0.2455	0.1488	0.0692	0.1086
R^2^	0.8106	0.8649	0.9091	0.8643	0.8792	0.8928
Adjusted R²	0.6402	0.7433	0.8273	0.7421	0.7705	0.7964

***Significant at *p* < 0.05; **significant at 0.05 ≤ *p* < 0.1; *significant at *p* ≥ 0.1.

(1) DPPH radical scavenging = + 70.5056–7.30098 A + 12.3746B + 2.06483 C-3.7625AB-2.6875AC + 1.1375BC-10.0212A^2^-8.60701B^2^-2.70267C^2^.

(2) Reducing power = 0.762378–0.0268893 A + 0.0765043B + 0.063587 C-0.0105AB-0.00325AC + 0.024BC -0.139705A^2^ -0.106648B^2^ -0.0753583C^2^.

According to the obtained results (Table 4; Fig. 3), the effect of time and substrate concentration on the reducing power of the hydrolysate was significant (*p* < 0.05), and the reducing power increased with increasing time, which caused more hydrolysis and production of peptides with high electron-donating ability. The high concentration of the substrate (optimal concentration) caused an increase in the reducing power. However, in concentrations higher than the optimal value, no significant increase was observed (*p* > 0.05) because the high concentration of the substrate may inhibit the production of the protease enzyme, which was consistent with the research of Fakhfakh et al.^36^. In relation to DPPH radical inhibition, increasing the temperature and time (Fig. 3) to the optimal value led to hydrolysate production with the highest inhibitory effect, which converted free radicals into stable compounds. Increasing the temperature more than the optimal level caused a decrease in enzyme activity. Huang et al.^37^ stated that fermentation temperature plays an important role in determining antioxidant activity. With hydrolysis over a more extended time, peptides may be hydrolyzed or consumed by bacteria as an energy source.

Regarding the antimicrobial activity, the highest inhibition against *S. aureus*, *E. coli*,* L. monocytogenes*, and *C. perfringens* was obtained at 78%, 62.7%, 85%, and 87.7%, respectively. According to the analysis of variance mentioned in Table 5, the effect of time, temperature, and variables A^2^ and B^2^ on the inhibition of *S. aureus* was significant (*p* < 0.05), among which A^2^ had a more significant effect. R^2^ of 0.9091 for this quadratic model (Eq. 3) showed the excellent ability of the proposed model to predict independent variables on the inhibition of *S. aureus.* A polynomial equation (quadratic model) was proposed for the inhibition of *E. coli* (Eq. 4). The coefficient of determination (R^2^) of 0.8643 showed that the model could explain 86% of the variations in the inhibitory effect of SPH against *E. coli.* According to the p-value of the quadratic model terms (Eq. 5) and R^2^ of 0.8792, time, temperature, and variables A^2^, B^2^, and C^2^ had statistically significant effects on the inhibitory effect of SPH against *L. monocytogenes*. Due to the analysis of variance results, R^2^ of 0.8928 demonstrated that the proposed model can well explain the effect of independent variables against *C. perfringens*.

(3) *S. aureus* inhibition = + 69.2652–11.1951 A + 15.1311B + 4.29781 C-8.15AB-2.35AC + 0.025BC-17.3741A^2^-11.9647B^2^-5.83057C^2^.

(4) *E. coli* inhibition = + 59.3089–8.09879 A + 10.615B + 4.78142 C-6.4AB-1.425AC-16.1956A^2^-10.6625B^2^-5.99557C^2^.

(5) *L. monocytogenes* inhibition = + 78.2311–10.418 A + 14.9587B + 5.47436 C-6.225AB-0.8AC-0.25BC-22.3774A^2^-14.0866B^2^-9.84396C^2^.

(6) *C. perfringens* inhibition = + 78.3746–7.51767 A + 15.3561B + 8.51145 C-4.462AB-3.3375AC-1.1625BC-18.9232A^2^-14.4508B^2^-8.67019 C.

According to the obtained results, the SPH had an antimicrobial effect. Independent variables significantly affected the antimicrobial activity of hydrolysate (Fig. 3). By increasing the time and determining the optimal temperature for the growth of *B. coagulans* and enzyme activity, the degree of hydrolysis increased. Antimicrobial peptides typically consist of less than 50 amino acids, approximately half of which are hydrophobic^38^. In addition, these peptides had a low molecular weight, usually less than 10 kDa^39^. The antimicrobial activity of peptides was very different depending on the type of amino acid, structure, length of peptides, and their sequence^40^. Amino acids such as tryptophan and arginine play an essential role in determining the antimicrobial properties of peptides^41^.

In the model validation, the optimal conditions suggested by the software were temperature 34.93 °C, time 31.34 h, and substrate concentration 4.92%. Under these conditions, DPPH radical inhibition and reducing power were 77.75% and 0.784, respectively. The inhibitory effect of SPH against *S. aureus*, *E. coli*, *L. monocytogenes*, and *C. perfringens* was 77.59%, 65.93%, 84.38%, and 85.69%, respectively. In the optimal conditions mentioned by the model, fermentation was carried out, and DPPH radical inhibition and iron ion reducing power were obtained, 79.26% and 0.758 (absorbance at 700 nm), respectively. The inhibitions of *S. aureus*, *E. coli*, *L. monocytogenes*, and *C. perfringens* were 78.28%, 63.68%, 80.43%, and 87.34%, respectively. These results showed the model’s ability to investigate the effect of independent variables on sesame protein hydrolysate’s antioxidant and antimicrobial activity.

### Antioxidant activity of the optimal treatment in different concentrations

#### Reducing power

**Fig. 4:**
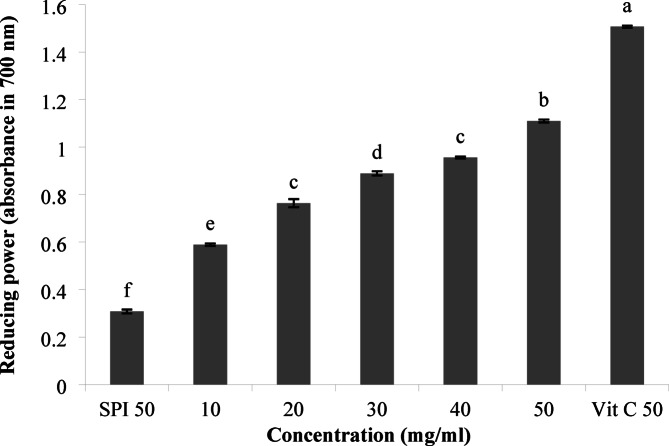
Ferric ion reducing power of the hydrolysate at different concentrations. Data values were performed in triplicate (Mean ± SD).

The reducing power is the ability of the protein hydrolysate to donate electrons. The absorbance at 700 nm shows the high reducing power of the antioxidant composition. As represented in Fig. 4, increasing the hydrolysate concentration increased the reducing power significantly (*p* < 0.05). All samples had lower reducing power than vitamin C, but the concentration of 50 mg/mL exhibited the highest reducing power compared to lower concentrations. Our results were consistent with those of Zhang et al.^42^. who investigated peanut protein hydrolysis by *Bacillus subtilis* and reported that the reducing power increased significantly with increasing concentration, which could be due to the production of low molecular weight bioactive peptides containing antioxidant amino acids. It was stated that the increase in the reducing power of the protein hydrolysate could be due to the increased production of electron-donating peptides^43^. Furthermore, the reducing power depends on the type of amino acids, such as tryptophan, tyrosine, methionine, histidine, and lysine, in the peptide chain and their sequence. On the other hand, protease activity during fermentation has an important role in determining the antioxidant properties of the peptide^44^.

#### DPPH radical inhibition

**Fig. 5:**
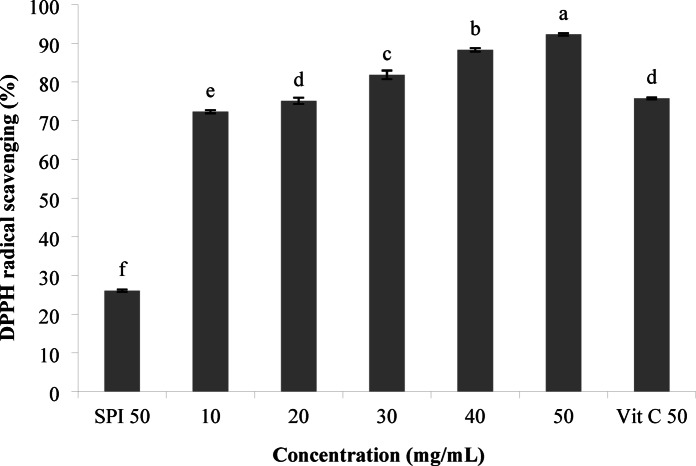
DPPH radical inhibition of the hydrolysates at different concentrations. Data values were performed in triplicate (Mean ± SD).

DPPH radical inhibition was used to evaluate the antioxidative potential of SPH at different concentrations, which had the highest absorbance at the wavelength of 517 nm. The results are shown in Fig. 5, DPPH radical inhibition activity increased with increasing concentration. Among different concentrations, the concentration of 50 mg/mL of hydrolysate showed the highest percentage of radical inhibition, which had a higher inhibitory activity compared to vitamin C. Our findings were in agreement with the research reported by Jemil et al.^21^ who stated that DPPH radical scavenging activity increased with increasing protein hydrolysate concentration. Therefore, the DPPH radical scavenging activity of hydrolysate obtained from fermentation by *Bacillus subtilis* was dose-dependent. Abd Rashid et al.^45^ reported that the higher DPPH radical inhibition ability of the fish protein hydrolysate in different concentrations depends on the type of protein substrate used in the fermentation process and the mechanism of action of proteolytic enzymes produced by different strains to produce peptides with different sizes. Zhi et al.^8^ reported that antioxidant activity depends on the amount and position of aromatic, acidic, and basic amino acids.

### Scanning electron microscopy

**Fig. 6:**
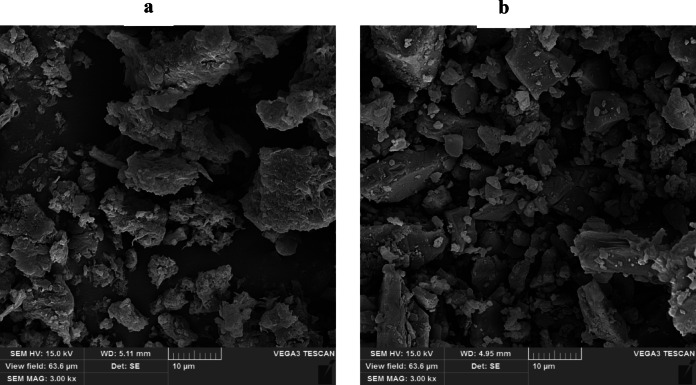
Scanning electron microscopy (SEM) of sesame meal protein isolate (**a**), and sesame meal protein hydrolysate (**b**).

Electron microscopy was performed to evaluate the microstructure of the protein and hydrolysate. According to Fig. 6, it was observed that the protein consists of heterogeneous, compact, and organized spherical particles^46^. The protein hydrolysate has formed smaller particles, indicating that the fermentation was able to hydrolyze the protein into a lower molecular weight peptide. The difference in morphological characteristics between protein isolate and protein hydrolysate can be attributed to the enzymatic hydrolysis of protein by proteases produced during the fermentation process, which has led to the breakdown of the protein molecular skeleton^23^.

#### FTIR spectroscopy

**Fig. 7:**
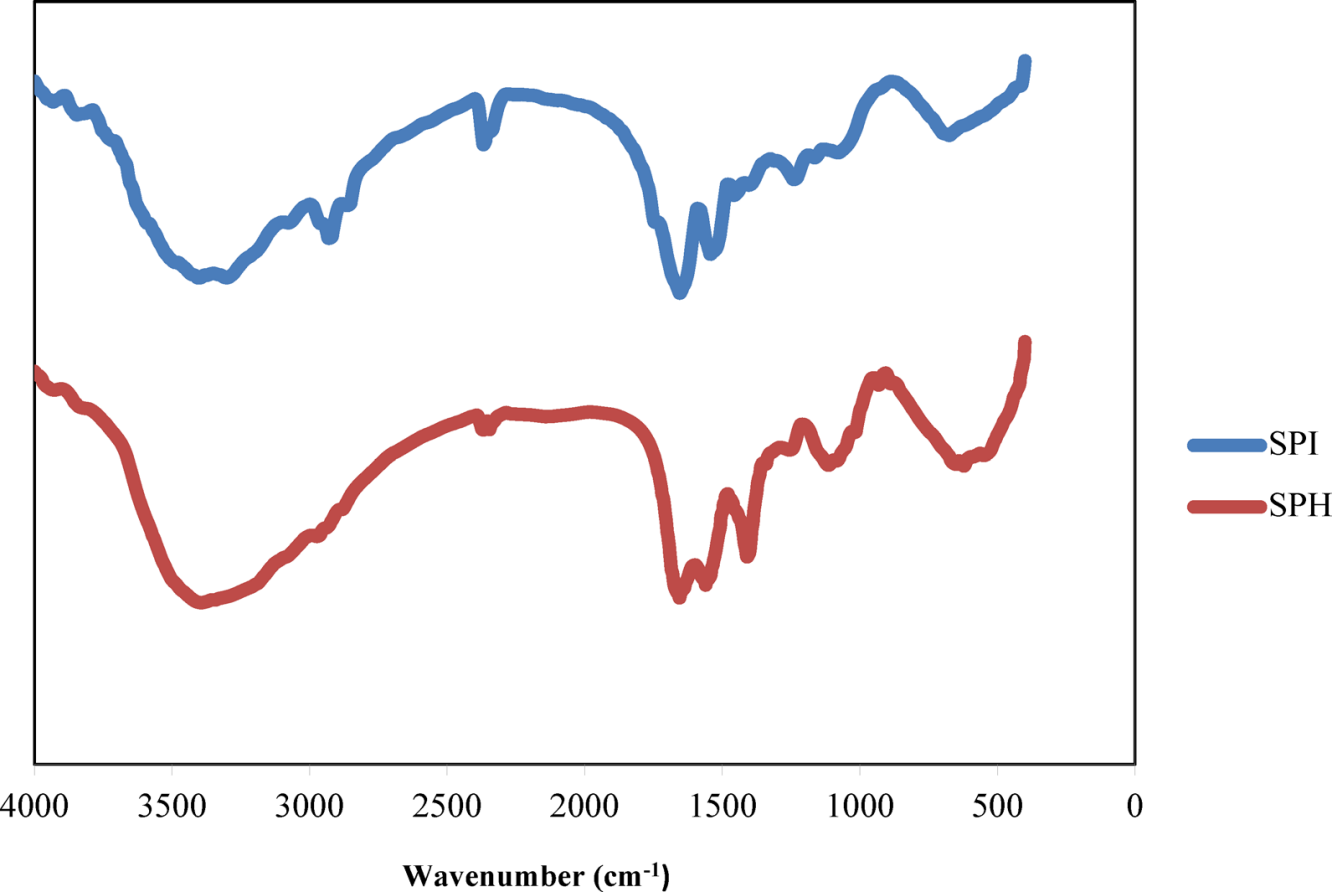
FTIR spectrum of sesame meal protein isolate and sesame meal protein hydrolysate.

The FTIR spectra of SPI and SPH (hydrolysis with *B. coagulans*) are given in Fig. 7. Sesame protein isolate (SPI) and sesame protein hydrolysate (SPH) showed some peaks in the regions 3394–3401, 2900, 1654, 1541–1560, and 1400–1457 cm^− 1^, which were correlated to amides A, B, amides I, II, and III respectively. Amide A showed O-H stretching and N-H stretching with hydrogen bonding. Stretching vibrations of the C = O groups or C = C in alkenes were related to amide I. Amide II showed C-N stretching vibration or N-H bending. The peak that appeared at 2900 cm^− 1^ was related to the C-H vibration of alkane^47,48^. According to the formation of different peaks and the creation of different functional groups, it can be concluded that *B. coagulans* has caused protein hydrolysis by producing protease.

## Conclusion

In this study, sesame protein, after extraction, was used to produce fermented hydrolysate by *B. coagulans*. According to the results, fermentation hydrolysis depends on factors such as fermentation temperature, time, type, and substrate concentration. Sesame seeds are a potential source of protein. Amino acid composition is an important parameter in the bioactivity properties of fermented peptides. All essential amino acids for sesame protein were recorded by HPLC. The results showed that *B. coagulans* had high proteolytic activity that caused the hydrolysis of sesame protein and produced hydrolysates with high antioxidant and antimicrobial capabilities. Based on the findings of this research, sesame meal protein hydrolysate can replace chemical preservatives and minimize their harmful effects.

## Data Availability

The datasets generated during this study are available from the corresponding author upon request (email: [khomeiri@gau.ac.ir]).

## References

[1] Salaheen, S., Peng, M. & Biswas, D. Replacement of conventional antimicrobials and preservatives in food production to improve consumer safety and enhance health benefits. *Microbial Food Saf. Preserv. Tech.* 311–314 (2015).

[2] Zhang, S., Luo, L., Sun, X. & Ma, A. Bioactive peptides: a promising alternative to chemical preservatives for food preservation. *J. Agric. Food Chem.* **69**, 12369–12384 (2021). 34649436 10.1021/acs.jafc.1c04020

[3] Tkaczewska, J. Peptides and protein hydrolysates as food preservatives and bioactive components of edible films and coatings-A review. *Trends Food Sci. Technol.* **106**, 298–311 (2020).

[4] Bouarab Chibane, L., Degraeve, P., Ferhout, H., Bouajila, J. & Oulahal, N. Plant antimicrobial polyphenols as potential natural food preservatives. *J. Sci. Food Agric.* **99**, 1457–1474 (2019). 30206947 10.1002/jsfa.9357

[5] Xu, X. et al. Synthetic phenolic antioxidants: Metabolism, hazards and mechanism of action. *Food Chem.* **353**, 129488 (2021). 33714793 10.1016/j.foodchem.2021.129488

[6] Lorenzo, J. M. et al. Bioactive peptides as natural antioxidants in food products–A review. *Trends Food Sci. Technol.* **79**, 136–147 (2018).

[7] Contesini, F. J., Melo, R. R. D. & Sato, H. H. An overview of *Bacillus* proteases: from production to application. *Crit. Rev. Biotechnol.* **38**, 321–334 (2018). 28789570 10.1080/07388551.2017.1354354

[8] Zhi, T. et al. Novel antioxidant peptides from protein hydrolysates of scallop (*Argopecten irradians*) mantle using enzymatic and microbial methods: Preparation, purification, identification and characterization. *LWT - Food Sci. Technol.* **164**, 113636 (2022).

[9] Jemil, I. et al. Novel bioactive peptides from enzymatic hydrolysate of sardinelle (*Sardinella aurita*) muscle proteins hydrolysed by *Bacillus subtilis* A26 proteases. *Food Res. Int.* **100**, 121–133 (2017). 28873670 10.1016/j.foodres.2017.06.018

[10] Hwang, C. F., Chen, Y. A., Luo, C. & Chiang, W. D. Antioxidant and antibacterial activities of peptide fractions from flaxseed protein hydrolysed by protease from *Bacillus altitudinis* HK02. *Int. J. Food Sci. Technol.* **51**, 681–689 (2016).

[11] Jemil, I. et al. Peptidomic analysis of bioactive peptides in zebra blenny (*Salaria basilisca*) muscle protein hydrolysate exhibiting antimicrobial activity obtained by fermentation with *Bacillus mojavensis* A21. *Process. Biochem.* **51**, 2186–2197 (2016).

[12] Montserrat-de la Paz, S. et al. Antioxidant and anti-inflammatory properties of bioavailable protein hydrolysates from lupin-derived agri-waste. *Biomol* **11**, 1458 (2021). 10.3390/biom11101458 PMC8533297 34680091

[13] El Khier, M. K. S., Ishag, K. E. A. & Yagoub, A. A. Chemical composition and oil characteristics of Sesame seed cultivars grown in Sudan. *Res. J. Agric. Sci.* **4**, 761–766 (2008).

[14] Chatterjee, R., Dey, T. K., Ghosh, M. & Dhar, P. Enzymatic modification of Sesame seed protein, sourced from waste resource for nutraceutical application. *Food Bioprod. Process.* **94**, 70–78 (2015).

[15] Lu, X., Zhang, L., Sun, Q., Song, G. & Huang, J. Extraction, identification and structure-activity relationship of antioxidant peptides from sesame (*Sesamum indicum L.*) protein hydrolysate. *Food Res. Int.* **116**, 707–716 (2019). 30716998 10.1016/j.foodres.2018.09.001

[16] American Association of Cereal Chemists. *Approved Methods Committee, Approved Methods of the American Association of Cereal Chemists* (AACC, 2000).

[17] Jiang, L., Wang, B., Li, B., Wang, C. & Luo, Y. Preparation and identification of peptides and their zinc complexes with antimicrobial activities from silver carp (*Hypophthalmichthys molitrix*) protein hydrolysates. *Food Res. Int.* **64**, 91–98 (2014). 30011734 10.1016/j.foodres.2014.06.008

[18] Bah, C. S., Carne, A., McConnell, M. A., Mros, S. & Bekhit, A. E. D. A. Production of bioactive peptide hydrolysates from deer, sheep, pig and cattle red blood cell fractions using plant and fungal protease preparations. *Food Chem.* **202**, 458–466 (2016). 26920319 10.1016/j.foodchem.2016.02.020

[19] Tavallaie, S., Khomeiri, M., Mousivand, M., Maghsoudlou, Y. & Hashemi, M. Starches from different sources hydrolysis using a new thermo-tolerant amylase complex produced by *Bacillus subtilis* T41a: characterization and efficiency evaluation. *LWT - Food Sci. Technol.* **112**, 108218 (2019).

[20] Aguilar-Toalá, J. E. et al. Assessment of multifunctional activity of bioactive peptides derived from fermented milk by specific *Lactobacillus plantarum* strains. *J. dairy. sci.* **100**, 65–75 (2017). 27865495 10.3168/jds.2016-11846

[21] Jemil, I. et al. Functional antioxidant and antibacterial properties of protein hydrolysates prepared from fish meat fermented by *Bacillus subtilis* A26. *Process. Biochem.* **49**, 963–972 (2014).

[22] Bougatef, A. et al. Purification and identification of novel antioxidant peptides from enzymatic hydrolysates of sardinelle (*Sardinella aurita*) by-products proteins. *Food Chem.* **118**, 59–565 (2010).

[23] Chen, G. et al. Preparation, characterization and the in vitro bile salts binding capacity of celery seed protein hydrolysates via the fermentation using *B. subtilis*. *LWT - Food Sci. Technol.* **117**, 108571 (2020).

[24] Raei, P., Khomeiri, M., Mahounak, A. S., Moayedi, A. & Kashiri, M. Characterization of bacterial Cellulose/PVA composite films incorporated with Sesame meal protein hydrolysate: Physicochemical, Antimicrobial, and antioxidant properties. *Appl. Food Res.* 100727 (2025).

[25] Achouri, A., Nail, V. & Boye, J. I. Sesame protein isolate: Fractionation, secondary structure and functional properties. *Food Res. Int.* **46**, 360–369 (2012).

[26] Yamauchi, K., Samanya, M., Seki, K., Ijiri, N. & Thongwittaya, N. Influence of dietary Sesame meal level on histological alterations of the intestinal mucosa and growth performance of chickens. *J. Appl. Poult. Res.* **15**, 266–273 (2006).

[27] Makinde, F. M. & Akinoso, R. Comparison between the nutritional quality of flour obtained from raw, roasted and fermented sesame (*Sesamum indicum L.*) seed grown in Nigeria. *Acta Sci. Pol. Technol. Aliment.* **13**, 309–319 (2014). 24887946 10.17306/j.afs.2014.3.9

[28] Onsaard, E. Sesame proteins. *Int Food Res J*. **19**, 1287–1295 (2012).

[29] Nweke, F. N., Ubi, B. E. & Kunert, K. J. Determination of proximate composition and amino acid profile of Nigerian Sesame (*Sesamum indicum L.*) cultivars. *Niger J. Biotechnol.* **23** (2011).

[30] Cruz-Casas, D. E. et al. Bioactive protein hydrolysates obtained from Amaranth by fermentation with lactic acid bacteria and *Bacillus* species. *Heliyon* **9** (2023). 10.1016/j.heliyon.2023.e13491 PMC9950839 36846651

[31] Datta, S. & Roy, A. Antimicrobial peptides as potential therapeutic agents: a review. *Int J Pept Res Ther*. **27**, 555–577 (2021).

[32] Moayedi, A., Hashemi, M. & Safari, M. Valorization of tomato waste proteins through production of antioxidant and antibacterial hydrolysates by proteolytic *Bacillus subtilis*: optimization of fermentation conditions. *J Food Sci Technol*. **53**, 391–400 (2016). 26787958 10.1007/s13197-015-1965-2 PMC4711411

[33] Li, W. & Wang, T. Effect of solid-state fermentation with *Bacillus subtilis* Lwo on the proteolysis and the antioxidative properties of Chickpeas. *Int. J. Food Microbiol.* **338**, 108988 (2021). 33267968 10.1016/j.ijfoodmicro.2020.108988

[34] Zhu, Y. P., Fan, J. F., Cheng, Y. Q. & Li, L. T. Improvement of the antioxidant activity of Chinese traditional fermented Okara (*Meitauza*) using *Bacillus subtilis* B2. *Food Control*. **19**, 654–666 (2008).

[35] Shi, C., Zhang, Y., Lu, Z. & Wang, Y. Solid-state fermentation of corn-soybean meal mixed feed with *Bacillus subtilis* and *Enterococcus faecium* for degrading antinutritional factors and enhancing nutritional value. *J. Anim. Sci. Biotechnol.* **8**, 1–9 (2017). 28603613 10.1186/s40104-017-0184-2 PMC5465572

[36] Fakhfakh, N. et al. Total solubilisation of the chicken feathers by fermentation with a keratinolytic bacterium, *Bacillus pumilus* A1, and the production of protein hydrolysate with high antioxidative activity. *Process. Biochem.* **46**, 1731–1737 (2011).

[37] Huang, Y. H., Lai, Y. J. & Chou, C. C. Fermentation temperature affects the antioxidant activity of the enzyme-ripened sufu, an Oriental traditional fermented product of soybean. *J. Biosci. Bioeng.* **112**, 49–53 (2011). 21497549 10.1016/j.jbiosc.2011.03.008

[38] Shen, W. et al. From antimicrobial peptides to antimicrobial Poly (α-amino acid) s. *Adv. Healthc. Mater.* **7**, 1800354 (2018). 10.1002/adhm.201800354 29923332

[39] Zhu, X. et al. Characterization of antimicrobial activity and mechanisms of low amphipathic peptides with different α-helical propensity. *Acta Biomater.* **18**, 155–167 (2015). 25735802 10.1016/j.actbio.2015.02.023

[40] Pane, K. et al. Antimicrobial potency of cationic antimicrobial peptides can be predicted from their amino acid composition: application to the detection of cryptic antimicrobial peptides. *J. Theor. Biol.* **419**, 254–265 (2017). 28216428 10.1016/j.jtbi.2017.02.012

[41] Jeżowska-Bojczuk, M. & Stokowa-Sołtys, K. Peptides having antimicrobial activity and their complexes with transition metal ions. *Eur. J. Med. Chem.* **143**, 997–1009 (2018). 29232589 10.1016/j.ejmech.2017.11.086

[42] Zhang, Y. et al. Isolation and identification of an antioxidant peptide prepared from fermented peanut meal using *Bacillus subtilis* fermentation. *Int*. *J Food Prop*. **17**, 1237–1253 (2014).

[43] Callegaro, K., Welter, N. & Daroit, D. J. Feathers as bioresource: microbial conversion into bioactive protein hydrolysates. *Process. Biochem.* **75**, 1–9 (2018).

[44] Zhu, K., Zhou, H. & Qian, H. Antioxidant and free radical-scavenging activities of wheat germ protein hydrolysates (WGPH) prepared with alcalase. *Process. Biochem.* **41**, 1296–1302 (2006).

[45] Abd Rashid, N. Y. et al. Evaluation of antioxidant and antibacterial activities of fish protein hydrolysate produced from Malaysian fish sausage (*Keropok Lekor*) by-products by Indigenous *Lactobacillus casei* fermentation. *J. Clean. Prod.* **347**, 131303 (2022).

[46] Du, X. et al. pH-shifting formation of goat milk casein nanoparticles from insoluble peptide aggregates and encapsulation of Curcumin for enhanced dispersibility and bioactivity. *LWT - Food Sci. Technol.* **154**, 112753 (2022).

[47] Zheng, L. et al. Effects of *Bacillus* fermentation on the protein microstructure and anti-nutritional factors of soybean meal. *Lett. Appl. Microbiol.* **65**, 520–526 (2017). 28975646 10.1111/lam.12806

[48] Brahmi, Y. et al. Conformational study of protein interactions with hydrogen-passivated amorphous silicon surfaces: effect of pH. *Appl. Surf. Sci.* **423**, 394–402 (2017).

